# Brincidofovir (CMX001) for the Treatment of Severe Adenoviral Pneumonia in Kidney Transplant Recipient

**DOI:** 10.7759/cureus.5296

**Published:** 2019-08-01

**Authors:** Praveen Sudhindra, Bettina Knoll, Rajat Nog, Nandita Singh, Abhay Dhand

**Affiliations:** 1 Medicine, New York Medical College, Valhalla, USA; 2 Infectious Diseases, Westchester Medical Center, Valhalla, USA; 3 Nephrology, Westchester Medical Center, Valhalla, USA

**Keywords:** adenovirus, transplantation, cidofovir, viral pneumonia

## Abstract

Adenovirus causes significant morbidity and mortality in solid organ and hematological transplant recipients. Treatment of adenovirus infections includes supportive care, reduction of immune suppression, and in patients with severe disease, intravenous cidofovir. Brincidofovir (CMX001) is a lipid conjugate of cidofovir, with good oral bioavailability, no associated nephrotoxicity, and higher intracellular levels of the active drug compared to cidofovir. We describe a case of severe adenoviral pneumonia in an adult renal transplant recipient who was successfully treated with oral brincidofovir after developing renal insufficiency with intravenous cidofovir. Brincidofovir (CMX001) along with other supportive therapy, may offer an efficacious, safe, and well-tolerated treatment for severe adenoviral infections in solid organ transplant recipients.

## Introduction

Adenovirus causes significant morbidity and mortality in solid organ and hematological transplant recipients [1]. Infection can be acquired de novo, result from reactivation of latent infection, or be donor organ derived. The clinical manifestations are protean, depending on the age group and the type of transplantation [1]. Suggested treatment of adenovirus infections includes supportive care and the reduction of immune suppression. In patients with severe disease, cidofovir use may be considered but is not supported by prospective randomized controlled trials or FDA approved for this indication [2]. Brincidofovir (CMX001) is a lipid conjugate of cidofovir, with good oral bioavailability, no associated nephrotoxicity, and higher intracellular levels of the active drug compared to cidofovir [3]. It has demonstrated in-vitro activity against several double-stranded deoxyribonucleic acid (DNA) viruses and a five to >2500-fold more potent in-vitro activity against adenovirus when compared to its parent compounds [4]. We describe a case of severe adenoviral pneumonia and acute respiratory failure in an adult renal transplant recipient who was successfully treated with oral brincidofovir after developing significant nephrotoxicity from intravenous cidofovir therapy.

## Case presentation

A 52-year-old Caucasian man, who had undergone renal transplantation 12 years ago, was admitted in the month of January with a five-day history of progressively worsening non-productive cough, nasal congestion, sore throat, and fever. His past history was significant for polycystic kidney disease, systolic heart failure, diabetes mellitus, hypertension, and morbid obesity. Home medications included prednisone, tacrolimus, mycophenolate mofetil (MMF), warfarin, insulin glargine, aspirin, furosemide, and carvedilol.

On admission, he had a temperature of 38.7 degrees centigrade, a pulse of 103 beats per minute, blood pressure of 166/91 millimeters of mercury and oxygen saturation of 96% breathing ambient air. Laboratory data revealed a white blood cell count of 8,500 cells/mm^3^ (78% Neutrophils, 8% Lymphocytes, 12.5% Monocytes), hemoglobin of 13.5 g/dL, platelet count of 167,000 cells/mm^3^, creatinine of 1.4 mg/dL, blood urea nitrogen of 20 mg/dL, and tacrolimus level of 8.8 ng/mL. Chest roentgenogram (CXR) revealed a right lower lobe infiltrate. Intravenous ceftriaxone and azithromycin were initiated for presumed community-acquired pneumonia. A respiratory pathogen multiplex polymerase chain reaction (PCR) (BioFire, Salt Lake City, USA) performed on a nasopharyngeal swab was positive for Adenovirus. MMF was discontinued, and the tacrolimus dose was reduced by 25% to target a trough level of 5 ng/mL. One dose of intravenous immunoglobulin (IVIg) was administered on day three (0.4 g/kg body weight). On day four, he had persistent fevers, worsening transaminitis, and developed acute hypoxemic respiratory failure requiring mechanical ventilation. CXR revealed increased consolidation in the right lower lobe as well as new left retrocardiac opacity. The serum creatinine worsened to 2.43 mg/dL. Adenovirus was isolated from an endotracheal aspirate by shell vial culture, and plasma PCR for Adenovirus DNA revealed a viral load of 188,000 copies/mL (limit of quantitation- 190 copies/ml). Rests of the diagnostic tests were negative for bacterial, fungal, other viral, or any non-infectious etiologies.

**Figure 1:**
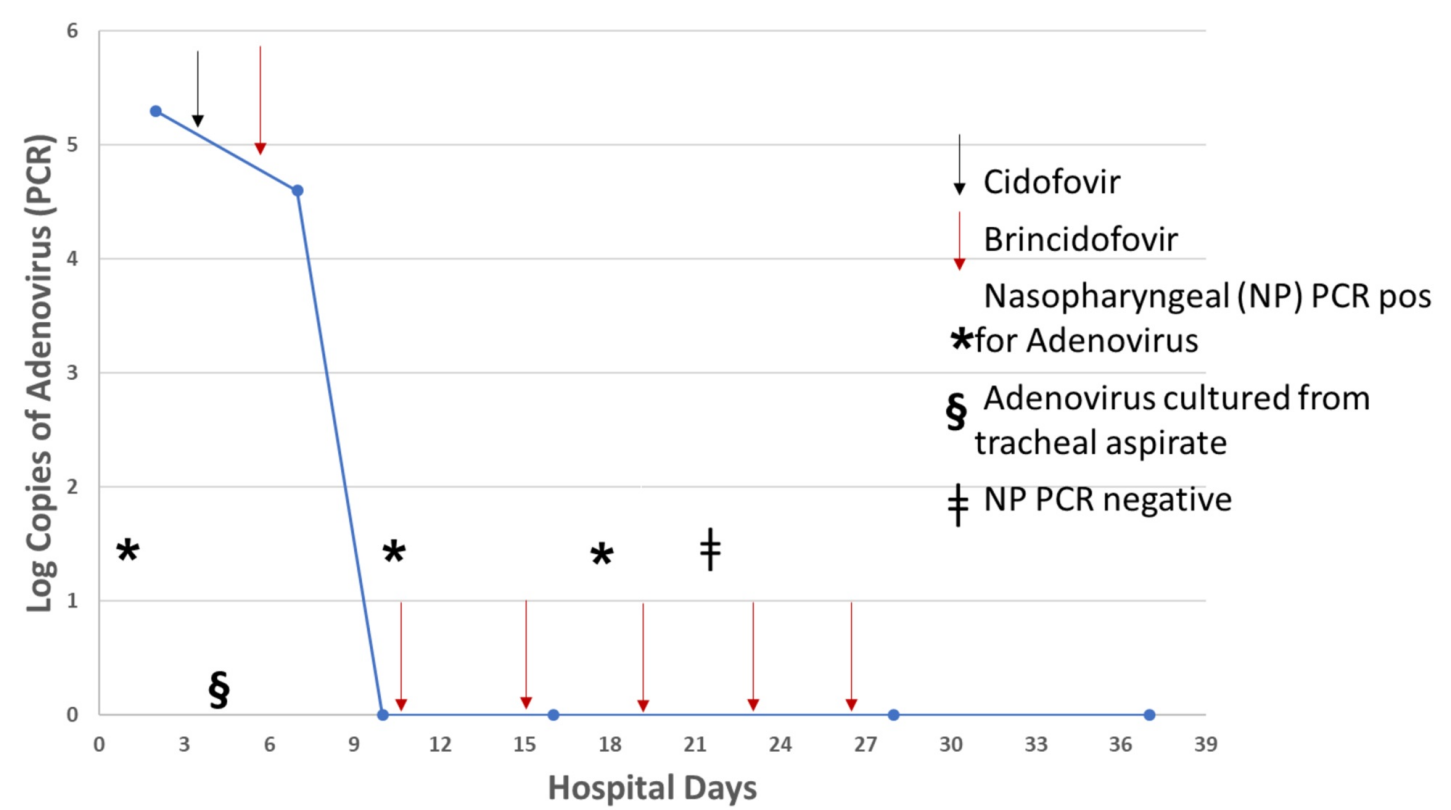
Diagnostic and treatment course during hospitalization

Treatment was started with one dose of intravenous cidofovir (5 mg/kg body weight) on hospital day five to treat severe adenoviral pneumonia. On day eight, he was started on 100 mg of oral brincidofovir, which was administered through the nasogastric tube at twice a week dosing. Brincidofovir was acquired from Chimerex through U.S. Food and Drug Administration (FDA) authorization under the Emergency Investigational New Drug program, and it was administered in accordance with the ethical standards laid down in the 1964 Declaration of Helsinki and its later amendments. During the rest of the hospital course, he received six more doses of brincidofovir. The patient clinically improved and was extubated on day 19. Treatment was discontinued on day 26 after full clinical recovery, and two consecutive plasma adenovirus PCR assays one week apart were negative. Renal function improved, with serum creatinine returning to baseline range by day 26. He was transferred to a rehabilitation facility on day 39 and was placed back on his admission immunosuppressive medications. The patient remained well on sixty-day follow up. Diagnostic tests, plasma viral loads, and treatment are summarized in Figure 1. 

## Discussion

Adenoviruses (AdV) are ubiquitous non-enveloped, double-stranded DNA viruses that typically cause self-limiting upper respiratory, conjunctival or gastrointestinal illnesses in immunocompetent hosts. AdV accounts for 10% of pneumonia in children, predominantly caused by serotypes 1-5, 7, 14, and 21 [1]. Serotypes 3, 4, 7, 14, and 21 have been isolated in outbreaks of respiratory disease in military recruits as well as civilian populations [5-8].

The overall incidence, risk factors for infection, and risk factors for progressive disease in hematopoietic stem cell transplant (HSCT) and solid organ transplant (SOT) recipients are listed in Table 1. 

**Table 1:** Important clinical characteristics of Adenovirus infection in Hematopoietic Stem Cell transplant (HSCT) and Solid Organ transplant (SOT) recipients

	HSCT recipients	SOT recipients
Incidence of infection	5%-47%	3.5% - 57.1%
Risk factors for higher incidence	Pediatric populations (31%-47% vs 13.6%) Allogeneic vs Autologous grafts (8.5%-30% vs 2%-12%) Receipt of T-cell depleted grafts (45% vs 11%) Acute Graft versus Host disease	Pediatric populations (except renal transplant); Lung, liver and multivisceral transplantation with the highest risk in intestinal transplant; Receipt of lymphocyte depleting antibodies; Sero-mismatch (Donor positive/recipient negative); First few months (~100 days) following transplantation
Progression to disseminated, fatal disease	In patients with severe lymphopenia (< 300 cells/mm^3 ^)	Unknown

In a case series of SOT, including kidney transplant recipients, the incidence of asymptomatic viremia ranged from 6.5-8.3% [9]. While viremia can often be transient and asymptomatic, the risk factors predicting progression to disease remain poorly defined. Treatment for acute rejection, low absolute lymphocyte count at the time of viremia, isolation/detection of virus from multiple sites, prolonged viremia as well as higher initial viral loads may all predispose to end-organ involvement and AdV disease [10]. The most common manifestations in renal allograft recipients include hemorrhagic cystitis (serotypes 11, 34, 35) and allograft nephritis [9,10]. Pneumonia is less common, usually manifesting as a part of disseminated disease, with up to 17% overall mortality [9-12].

The diagnosis of adenovirus disease can be challenging in immunocompromised populations. There is evidence that the virus can establish latency following acute infection and can be intermittently shed in tears and feces for prolonged periods [13,14]. PCR is highly sensitive and can be performed on peripheral blood, serum, cerebrospinal fluid (CSF), urine, as well as throat or nasopharyngeal swabs and by itself is not enough to make a diagnosis of adenoviral infection. Detection by PCR, along with the presence of attributable signs/symptoms and the absence of an alternative cause is required to make the diagnosis of AdV disease. Real-time PCR enables quantification and is useful in monitoring viral load, and consequently, the response to therapy. Tissue cultures can be used to isolate AdV (except for serotypes 40, 41) in human epithelial cell lines. However, this modality may not be practical in many clinical situations since it can take up to four weeks to isolate the virus. Viral cytopathic effects can be demonstrated by histopathologic examination of tissue. Other available diagnostic modalities, used less frequently, include rapid antigen detection, immunofluorescence assays (used primarily on respiratory specimens), enzyme immunoassays, latex agglutination and immune chromatography (stool samples). Serologic testing is of questionable significance in transplant recipients due to variability in immune responses [2,15].

Treatment of adenovirus infection in transplant recipients involves a reduction of immune suppression. No single strategy of optimizing immunosuppression is thought to be clearly superior in controlling infection as well as managing the risk of graft rejection. While antiviral agents appear to be of greatest benefit in severe disease, the data supporting their use comes largely from case reports and their overall contribution in controlling infection is unclear [1,2,15].

Cidofovir is a cytosine analog and an inhibitor of viral DNA polymerase and is the most widely used agent to treat severe adenoviral disease in stem cell transplant recipients [16]. Nephrotoxicity (30%-59% depending on the dose) and neutropenia can limit the utility of this drug, especially in renal transplant recipients in whom graft nephritis can occur commonly with adenoviral infection [15].

Brincidofovir (CMXOO1) is a lipid conjugate of cidofovir (hexadecyloxypropyl-cidofovir) with potent in-vitro activity against a number of double-stranded DNA viruses. The major advantages of brincidofovir include high oral bioavailability as well as the absence of nephrotoxicity. The drug achieves high intra-cellular concentrations by utilizing lipid uptake pathways in target cell membranes. It is then cleaved to yield cidofovir, which in turn is phosphorylated to produce the active drug cidofovir di-phosphate, a potent inhibitor of viral DNA synthesis. Renal toxicity of cidofovir is caused by the concentration of the drug in renal cells by the organic ion transporter, dopamine active transporter (hDAT- 1). Brincidofovir is not a substrate for this transporter and hence, does not cause nephrotoxicity [3].

Among solid organ transplant recipients, brincidofovir (CMXOO1) use for the treatment of adenoviral infection has been reported in two published studies in intestinal transplant recipients with gastrointestinal disease and one published study in a liver-kidney recipient [17-19]. The first study was a retrospective analysis among a multi-center cohort of eight pediatric and five adult patients with severe AdV disease who were refractory to or intolerant of other therapies. Among thirteen study subjects, there was only one solid organ transplant recipient, who had undergone intestinal transplantation. Nine patients (69.2%) had a virologic response at week eight, and the drug was found to be well tolerated [17]. In the second published study, brincidofovir was used to successfully treat adenoviral enteral infection in two intestinal transplant recipients [18]. In the third study, a liver-kidney transplant recipient who presented with a viral syndrome, had detection of adenovirus in blood and urine using a PCR, and no other end-organ involvement was successfully treated with brincidofovir for twelve weeks [19]. Doses and duration of treatment were variable in all in the studies.

## Conclusions

The diagnosis of adenovirus disease can be challenging in immunocompromised patients and requires evidence of adenoviral replication, presence of attributable signs/symptoms, and the absence of an alternative cause. While antiviral agents appear to be of greatest benefit in severe disease, the data supporting their use is limited, and their overall contribution in controlling the infection is unclear. Most commonly used antiviral agent is intravenous cidofovir, and its use can be limited by associated nephrotoxicity and neutropenia. We report a successful brincidofovir treatment of severe adenoviral disease presenting with pneumonia and acute respiratory failure in an adult kidney transplant recipient. Our report supports the in-vitro activity data and suggests that brincidofovir in addition to the reduction of immunosuppression may offer an efficacious, safe, and well-tolerated treatment for adenoviral disease in selected transplant recipients.
